# Publisher Correction: Giant photothermal nonlinearity in a single silicon nanostructure

**DOI:** 10.1038/s41467-020-19073-5

**Published:** 2020-10-07

**Authors:** Yi-Shiou Duh, Yusuke Nagasaki, Yu-Lung Tang, Pang-Han Wu, Hao-Yu Cheng, Te-Hsin Yen, Hou-Xian Ding, Kentaro Nishida, Ikuto Hotta, Jhen-Hong Yang, Yu-Ping Lo, Kuo-Ping Chen, Katsumasa Fujita, Chih-Wei Chang, Kung-Hsuan Lin, Junichi Takahara, Shi-Wei Chu

**Affiliations:** 1 Department of Physics, National Taiwan University, 1, Sec 4, Roosevelt Rd., 10617 Taipei, Taiwan; 2 Graduate School of Engineering, Osaka University, 2-1 Yamadaoka, Suita, Osaka, 565-0871 Japan; 3 Institute of Physics, Academia Sinica, 128, Sec. 2, Academia Rd., 11529 Taipei, Taiwan; 4 AIST-Osaka University Advanced Photonics and Biosensing Open Innovation Laboratory, AIST, 2-1 Yamadaoka, Suita, Osaka, 565-0871 Japan; 5 Institute of Photonic System, National Chiao Tung University, 301 Gaofa 3rd Road, 711 Tainan, Taiwan; 6 Institute of Imaging and Biomedical Photonics, National Chiao Tung University, 301 Gaofa 3rd Road, Tainan, 711 Taiwan; 7 Center for Condensed Matter Sciences, National Taiwan University, 1, Sec 4, Roosevelt Rd., 10617 Taipei, Taiwan; 8 Photonics Center, Graduate School of Engineering, Osaka University, 2-1 Yamadaoka, Suita, Osaka, 565-0871 Japan; 9 Molecular Imaging Center, National Taiwan University, 1, Sec 4, Roosevelt Rd., 10617 Taipei, Taiwan; 10 Brain Research Center, National Tsing Hua University, 101, Sec 2, Guangfu Road, 30013 Hsinchu, Taiwan

**Keywords:** Metamaterials, Silicon photonics, Nonlinear optics

Correction to: *Nature Communications* 10.1038/s41467-020-17846-6, published online 14 August 2020.

The original version of this Article contained an error in Fig. 4b, in which the *x*-axis label incorrectly read: “Time (s)”. The correct form of the *x*-axis label of Fig. 4b is: “Time (ns)”.

This has been corrected in both the PDF and HTML versions of the Article.

