# Supplementary material for: Giant photothermal nonlinearity in a single silicon nanostructure
Source: Nat Commun. 2020 Aug 14;11:4101. doi: 10.1038/s41467-020-17846-6 (PMC7427991; doi:10.1038/s41467-020-17846-6)
Supplement: Supplementary file 1 — Supplementary Information [file 41467_2020_17846_MOESM1_ESM.pdf]

Supplementary Information for:

Giant photothermal nonlinearity in a single silicon nanostructure

Duh and Nagasaki et al.

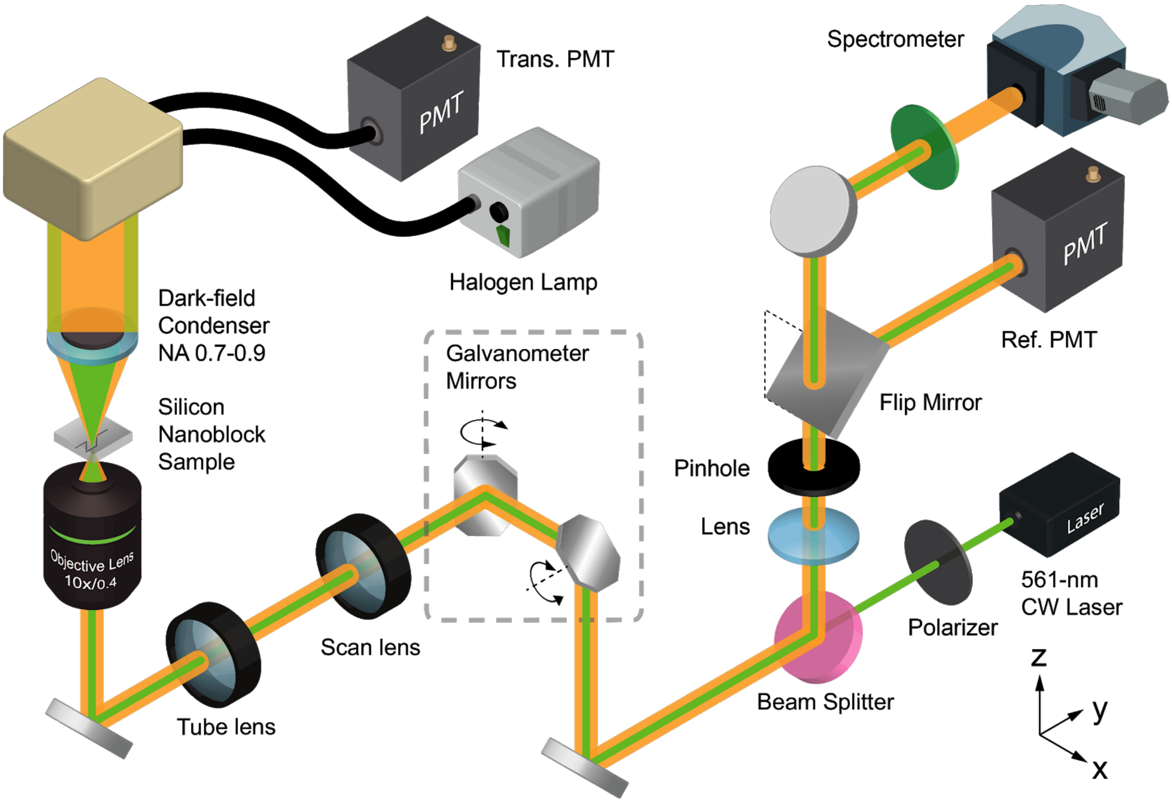

Supplementary Figure 1 | Dual function dark-field imaging system: lamp-illumination for single-nanostructure spectrum, and laser-scanning for nonlinearity characterization.

The orange beam path marks the optical path for scattering spectrum measurement. A broadband halogen lamp source illuminated silicon nanostructure through a dark-field condenser, and scattered light is collected with an objective. A set of galvo mirrors guide scattering from a specific nanoblock to a spectrometer. On the other hand, the green beam shows the dark-field laser scanning path for characterizing point spread function and single-nanoblock nonlinearity. In brief, a 561-nm continuous-wave laser is sent into the galvo scanner to achieve raster scan at focal plane of the objective. The forward scattered light is collected by the dark-field condenser, and delivered to a photomultiplier tube in the transmission path (trans PMT). For oil-immersed sample (Supplementary Figure 11), image is acquired by a photomultiplier tube in the reflection path (ref. PMT).

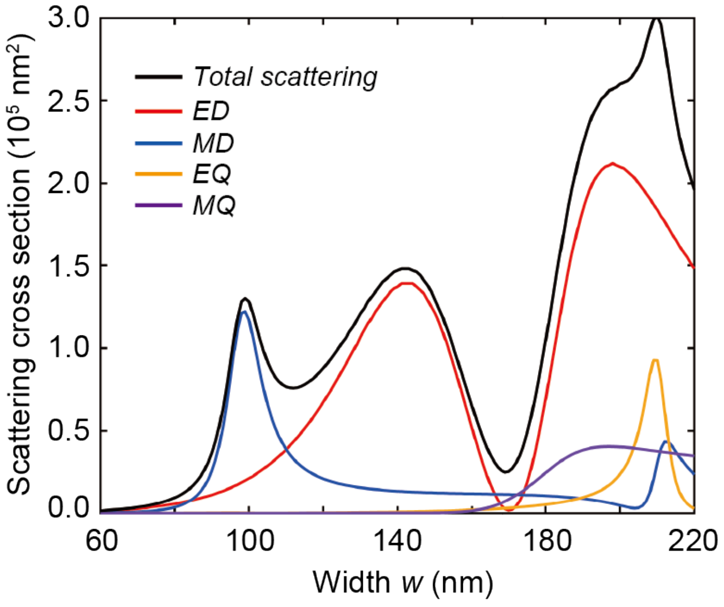

Supplementary Figure 2 | Multipole decomposition analysis of a Si nanoblock based on 561-nm excitation.

Scattering contributions of electric dipole (ED), magnetic dipole (MD), electric quadrupole (EQ), and magnetic quadrupole (MQ) versus nanoblocks' width. Higher-order poles are neglected due to their small contributions.

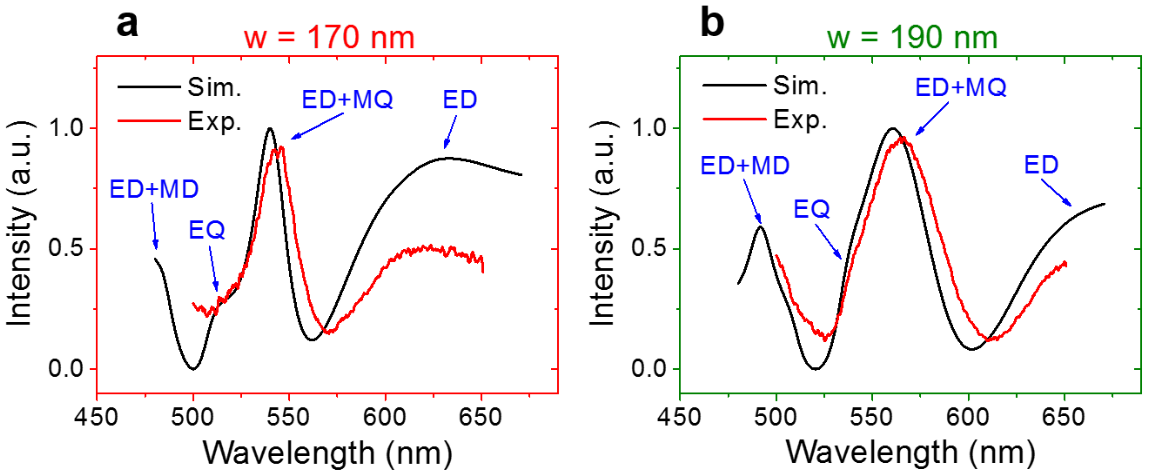

Supplementary Figure 3 | Scattering spectra of isolated nanoblocks: correlation between simulation and experiment.

Dark-field spectra of **a.** w = 170 nm and **b.** w = 190 nm. The black and red lines are simulation and experimental acquisition, respectively, revealing nice agreement between each other. In our experiment, excitation wavelength is 561-nm, which locates at a spectral valley of the 170-nm-width nanoblock (Fig. **a**) and at a spectral peak of the 190-nm-width nanoblock (Fig. **b**), respectively. Since Mie scattering spectrum red-shifts at elevated temperature, this explains the reason why the w =

170 nm nanoblock provides large positive nonlinearity (Fig. 2b), and w = 190 nm shows negative nonlinearity (Fig. 2c).

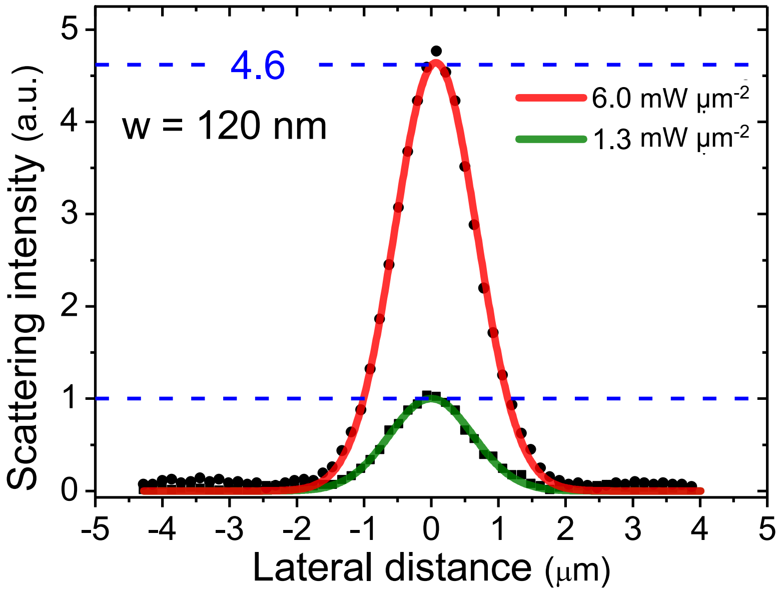

Supplementary Figure 4 | Intensity-dependent scattering profile for a linear silicon nanoblock ($w_x = w_y$ = 120 nm).

At low intensity (green curve, 1.3 mW μm^{-2}) and high intensity (red curve, 6.0 mW μm^{-2}), both PSFs exhibit Gaussian profile (black dots are experimental data). The ratio of peak values meets exactly the ratio of intensities (4.6 = 6.0 / 1.3), manifesting the linearity of microscope and detection system.

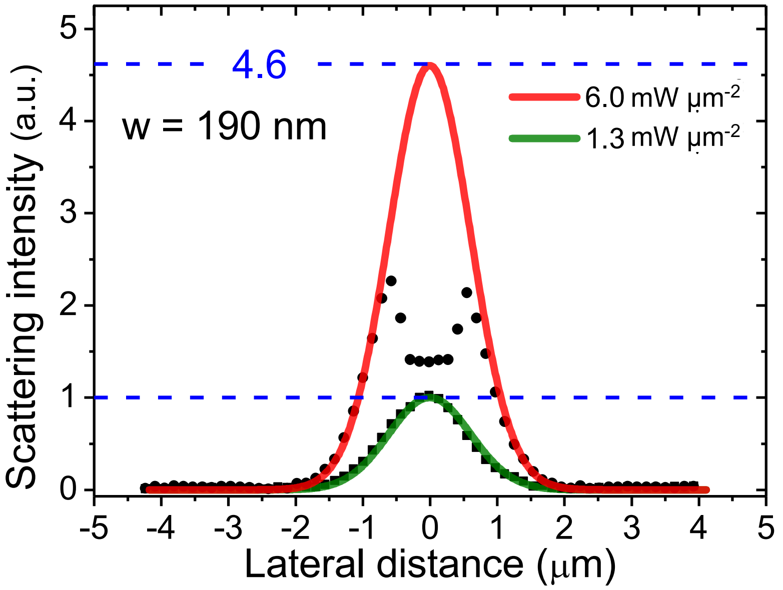

Supplementary Figure 5 | Intensity-dependent scattering profile for a nonlinear silicon nanoblock ($w_x = w_y$ = 190 nm)

At low intensity (1.3 mW μm^{-2}), the PSF shows a Gaussian profile (green curve), as expected. However, at high intensity (6.0 mW μm^{-2}), the measured PSF (black dots) drops in the center, significantly deviating from the extrapolated linear response (red curve, which is 4.6 times the green line), manifesting the existence of nonlinearity.

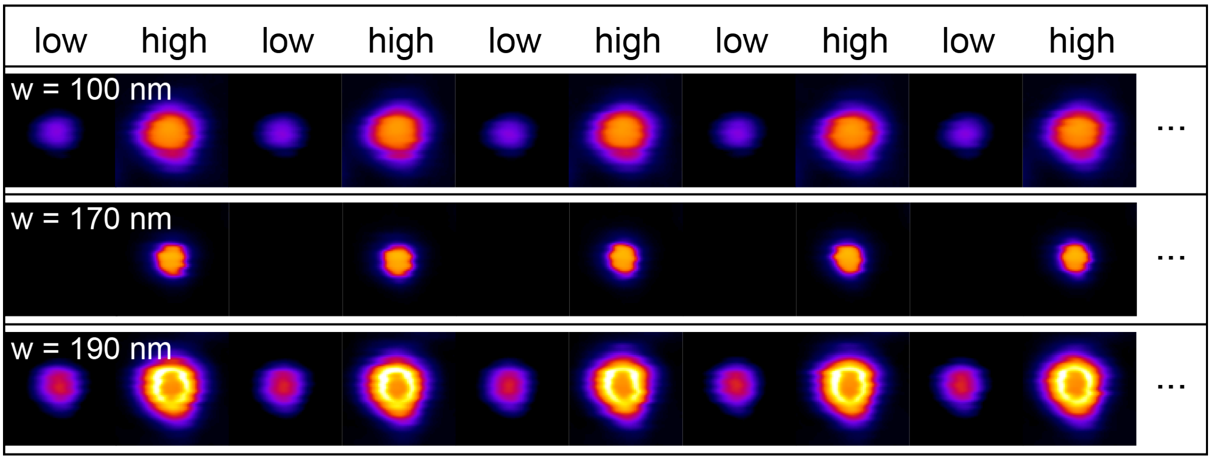

Supplementary Figure 6 | Reversibility and repeatability of nonlinear behaviors.

The PSF recovery during repetitive switching between low-intensity (1.3 mW μm^{-2}) and high-intensity (6.0 mW μm^{-2}) excitations, demonstrating the photothermal nonlinearity are reversible and repeatable.

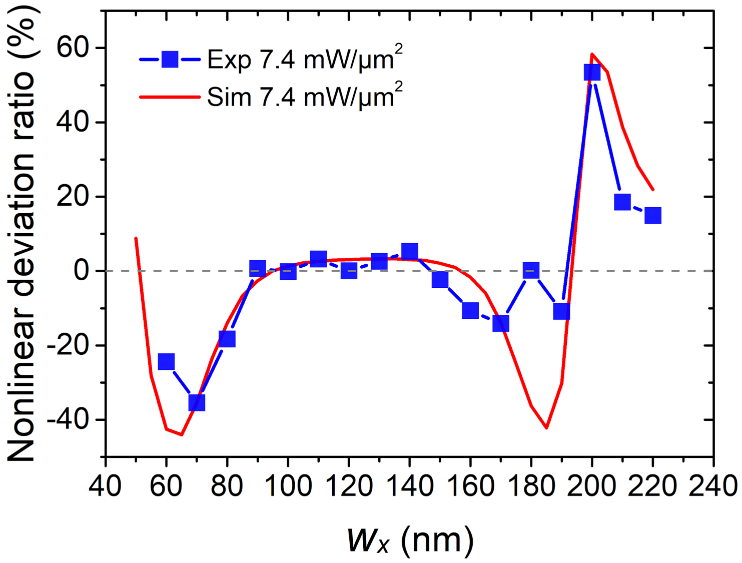

Supplementary Figure 7 | Additional confirmation of NDR between experiment and simulation.

Calculated nonlinearity deviation agrees with experiment for nanoblock with various w_x and fixed w_y=120 nm, as shown by a horizontal dashed line in Fig. 2f.

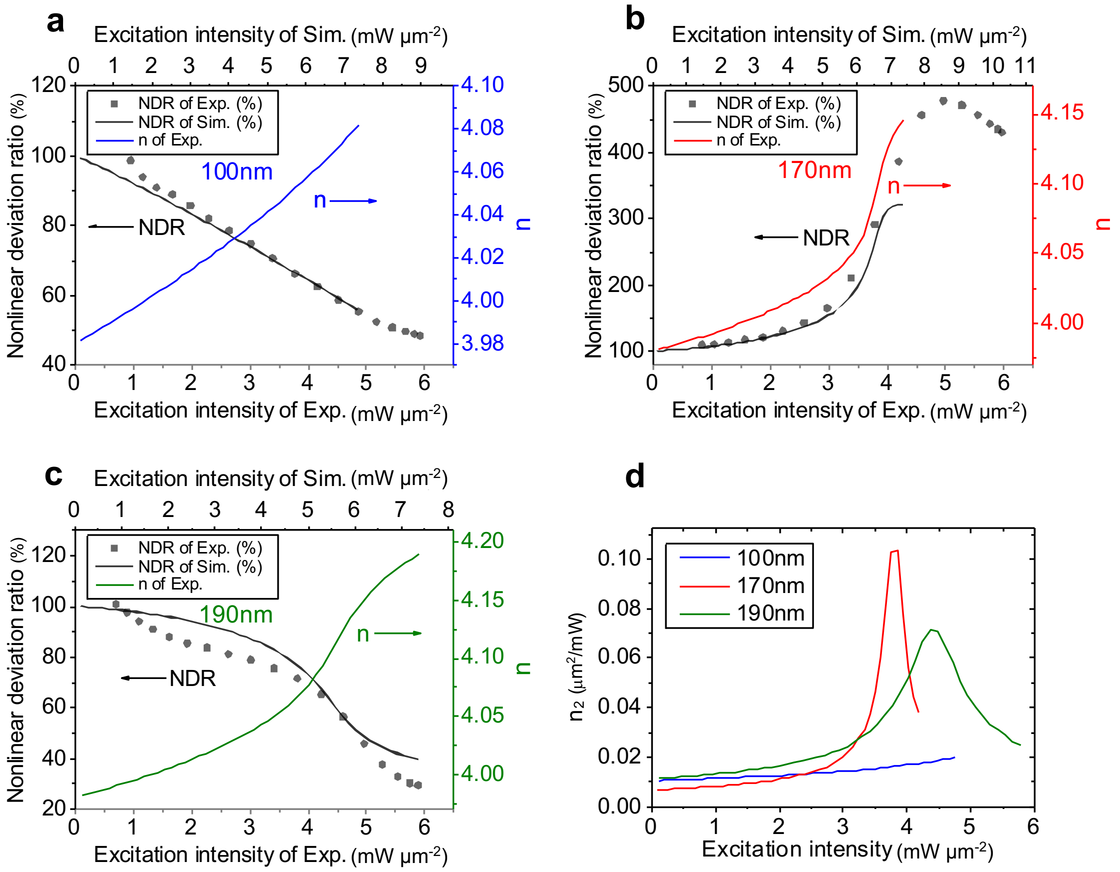

Supplementary Figure 8 | Derivation of nonlinear refractive index n_2 based on NDR in Fig. 2.

The experimental (grey dots) and simulated (black solid lines) NDRs for w = **a.** 100 nm, **b.** 170 nm, and **c.** 190 nm, respectively. The colored curves represent corresponding variation of refractive index versus excitation intensity. The detailed processes to derive these curves are listed below:

1. From thermal simulation (see method: "**Calculation of laser-induced temperature rise and relaxation**"), the temperature of each nanoblock at each excitation intensity is found.
2. Refractive index (n) of silicon at elevated temperature is determined through ellipsometry measurement and extrapolation (see Supplementary Figure 9).
3. The temperature- and intensity-dependent refractive index is taken into Mie theory calculation, to find out scattering intensity of a single silicon nanoblock at various excitation intensity.
4. Nonlinear deviation ratio (NDR = ΔS/S) is determined as the percentage deviation of measured scattering (ΔS) over extrapolated linear response (S) from the scattering at low excitation intensity.

d. The nonlinear indices of each nanoblock, extracted by differentiation of refractive index over excitation intensity. The nonlinear index reaches 0.1 $\mu m^2 mW^{-1}$, which is much larger than either Kerr or photothermal nonlinearity of bulk silicon.

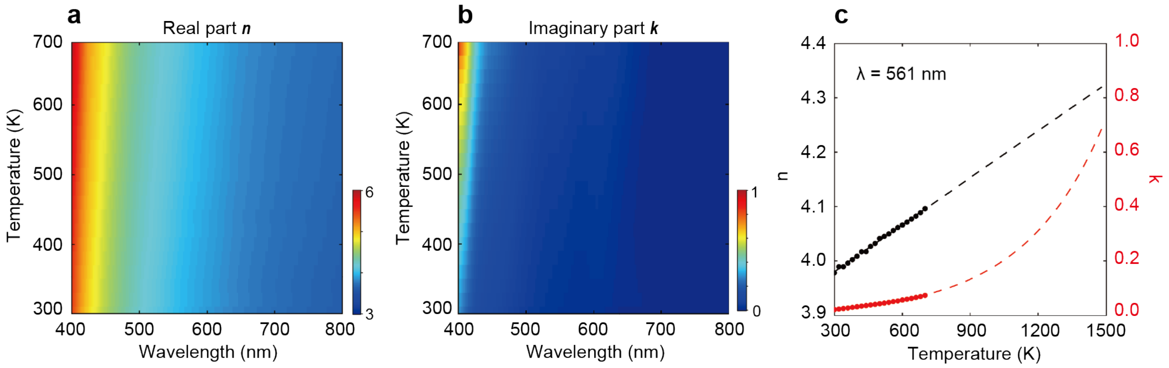

Supplementary Figure 9 | Temperature-dependent refractive index of Si by ellipsometry.

a. Real and **b.** imaginary part of silicon refractive index, measured in wavelength range of 400-800 nm, and temperature range of 300-700 K. **c,** Temperature-dependent refractive index at 561-nm (real part n: black dots; imaginary part k: red dots). The extrapolation (dashed lines) to 1500K is based on published equations (see Methods: Refractive index of silicon at elevated temperature).(*29*)

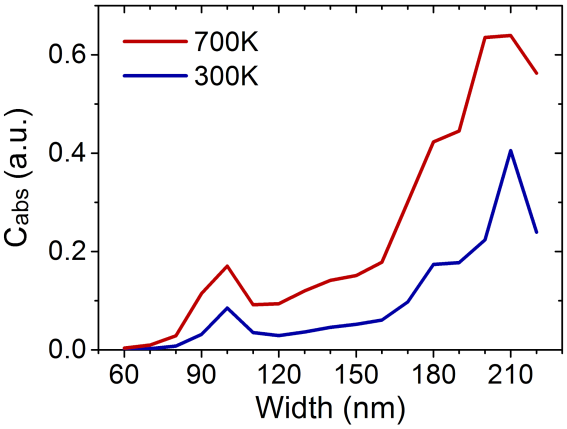

Supplementary Figure 10 | Absorption cross section(C_{abs}) (at 561 nm excitation) of each nanoblock at 300 K (blue) and 700 K (red).

There are three noteworthy points. First, similar to Fig. 1d, multiple peaks are found, but the peak positions of size-dependent absorption here are different from scattering in Fig. 1d. The absorption-scattering peak difference is common in the case of high-index nanostructure resonance due to the interaction of multiple poles. Second, the absorption cross sections of all poles increase with temperature. Therefore, an iterative calculation (see Methods) is necessary to derive correct temperature values under photothermal effect. Third, the MD absorption is relatively small, but the resulting temperature elevation (Fig. 3b) is comparable to that of ED and MQ, i.e. MD resonance seems to be an efficient heating source. Possible

explanation is that the Q-factor of MD resonance is relatively large, leading to efficient excitation. In addition, MD dominates in small nanoparticles (100 nm in this case), whose thermal capacitance is also small, resulting in large temperature increase.

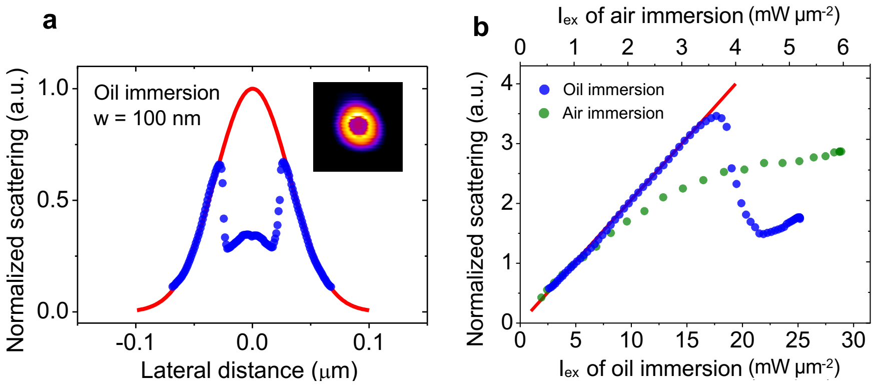

Supplementary Figure 11 | Very sharp nonlinearity by changing the immersion medium

a. Intensity profile of point-spread function (inset) from the w = 100 nm nanoblock that is immersed in oil, showing steep variation in the center. **b.** The corresponding scattering versus excitation intensity dependency, revealing surprisingly sharp nonlinear behavior. The green dots are nonlinear scattering with air immersion, i.e. data from Fig. 2a, for comparison. It is obvious that the nonlinear response is sensitive to the surrounding medium, and oil immersion provides much more steep variation that is potentially useful for high-contrast all-optical control with small power modification.

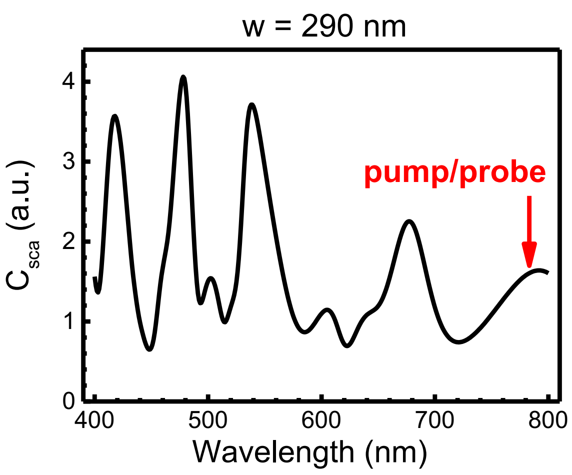

Supplementary Figure 12 | Spectrum of nanoblock for transient scattering measurement

Calculated scattering spectrum of a w = 290-nm nanoblock immersed in oil, manifesting its resonance with the 785-nm pump/probe laser.

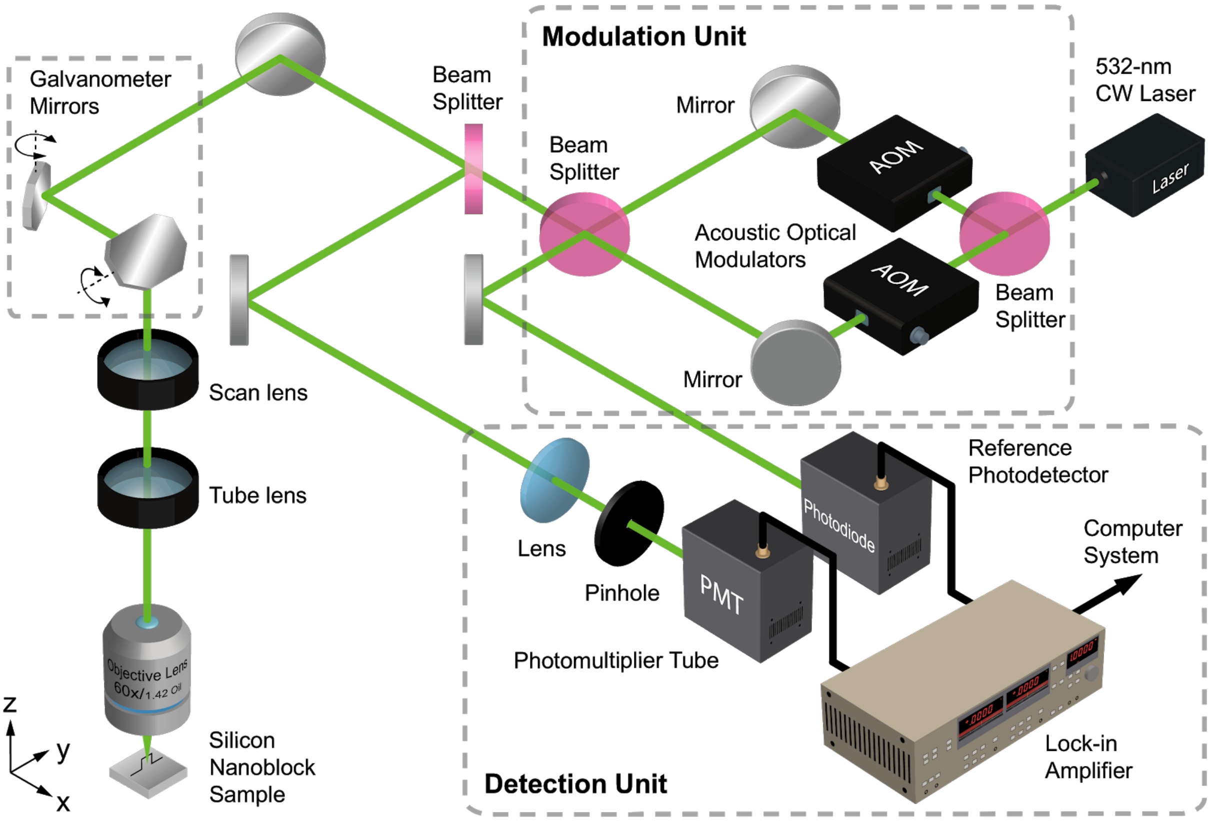

Supplementary Figure 13 | Setup of SAX super-resolution microscopy

Please refer to "Methods: Saturated excitation microscopy (SAX) to enhance resolution" for detailed description.
